# A Case of Asymptomatic Calcified Cerebral Emboli Harboring a Vulnerable Calcified Carotid Plaque: Diagnostic and Therapeutic Considerations

**DOI:** 10.7759/cureus.84860

**Published:** 2025-05-26

**Authors:** Koichi Iwasaki, Kazushi Kitamura, Hiroyo Yamahira, Hiroshi Hasegawa, Isao Sasaki

**Affiliations:** 1 Neurosurgery, Ainomiyako Neurosurgery Hospital, Osaka, JPN; 2 Cardiology, Ainomiyako Neurosurgery Hospital, Osaka, JPN

**Keywords:** asymptomatic, calcified cerebral emboli, preventative intervention, punctate intracranial calcifications, unstable carotid plaque

## Abstract

Punctate intracranial calcifications observed on non-contrast computed tomography (CT) as sequelae of calcified cerebral emboli (CCE) may be easily overlooked, particularly in asymptomatic patients. However, such findings can serve as critical indicators of underlying embolic sources, such as unstable or heavily calcified carotid plaques. Accurate recognition is essential, as timely therapy can significantly reduce the risk of future cerebrovascular events.

We report the case of an 84-year-old man with a past history of carotid artery stenting for symptomatic left internal carotid artery (ICA) stenosis performed five years earlier. At that time, no evidence of stenosis was observed in the right ICA. The patient remained asymptomatic, until non-contrast brain CT incidentally revealed multiple punctate calcific foci in the right cerebral hemisphere, exhibiting the characteristic "salted pretzel sign". Suspected newly proved CCE, subsequent diagnostic evaluations were performed to reveal a heavily calcified carotid plaque in the right ICA, suggestive of a potential embolic source. The patient subsequently underwent an uneventful carotid endarterectomy to prevent future ischemic events.

This case highlights the clinical significance of the accurate diagnosis of asymptomatic CCE and its underlying embolic source such as a high-risk carotid plaque. Furthermore, importance of optimal preventative intervention is emphasized to reduce the risk of subsequent ischemic insults.

## Introduction

Calcified cerebral emboli (CCE), once regarded as a rare etiology of ischemic stroke, is now increasingly recognized, accounting for approximately 2.7-5.9% of all acute cerebral infarctions [1-4]. Despite this, CCE often remains unrecognized or misinterpreted due to its subtle and non-specific radiologic features, posing both diagnostic and therapeutic challenges [1-4]. Radiographically, CCE typically appears on non-contrast CT as multiple, small (2-3 mm in diameter), hyperattenuating lesions. When these lesions are round or ovoid and resemble salt crystals, the characteristic pattern is colloquially referred to as the "salted pretzel sign" [5,6]. However, CCE may be easily overlooked, particularly in asymptomatic individuals, as it can mimic other intracranial pathologies such as old hemorrhage, vascular wall calcification, or sequelae of prior infections [3,7]. Although these punctate calcifications may not cause immediate clinical symptoms, they serve as critical markers of underlying embolic risk and may cause future cerebrovascular events [3,7,8]. This is especially significant when CCE is associated with embolic sources such as unstable, heavily calcified carotid plaques, which can substantially increase the risk of stroke [8,9].

We report here a rare case of asymptomatic CCE associated with an unstable and heavily calcified carotid plaque, emphasizing the diagnostic challenges and therapeutic considerations in managing such patients. While most reported cases of CCE present with clinical symptoms, our case is unique in that CCE was incidentally detected in an asymptomatic individual with a high-risk carotid plaque. To our knowledge, no previous reports have specifically addressed the diagnostic and therapeutic considerations for asymptomatic CCE in the context of high-risk carotid plaque.

## Case presentation

**Figure 1:**
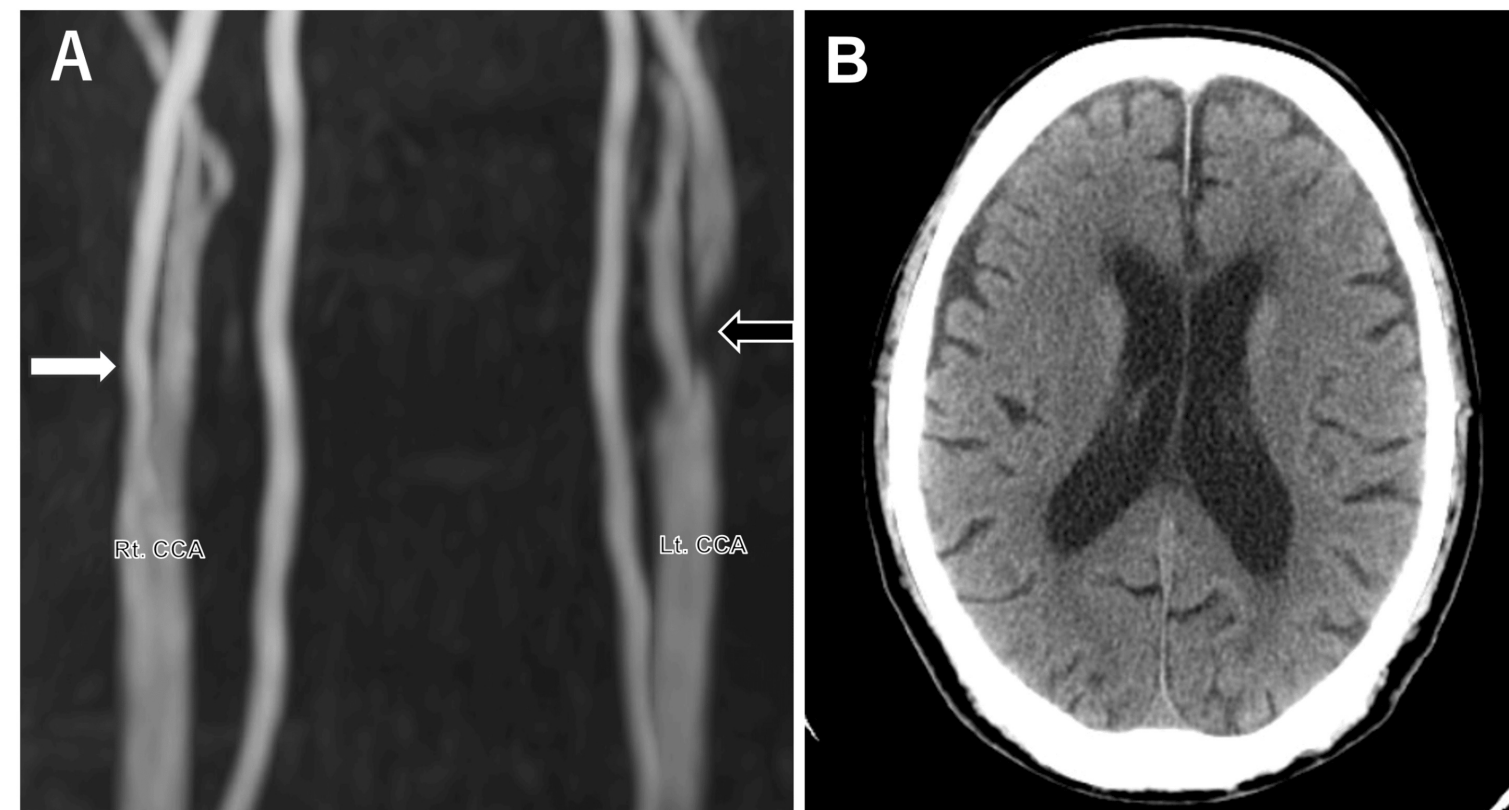
Radiological images at the initial intervention (A) Cervical MRA shows severe stenosis in the left ICA (black arrow), which was subsequently treated with carotid artery stenting. No stenosis is then identified in the right ICA (white arrow). (B) Non-contrast brain CT scan at that time identifies no evidence of low-density areas or cerebral calcifications. ICA: internal carotid artery, CCA: common carotid artery; MRA: magnetic resonance angiography; CT: computed tomography

An 84-year-old man underwent carotid artery stenting (CAS) for symptomatic severe stenosis of the left internal carotid artery (ICA) five years ago (Figure 1A). At the time of the intervention, no evidence of stenosis was observed in the right ICA (Figure 1A). Non-contrast computed tomography (CT) scan performed at that time revealed no cerebral calcifications or low-density regions (Figure 1B). The patient was subsequently maintained on antiplatelet therapy (aspirin, 100 mg/day) and, until representation, did not have the need to undergo any further imaging. Throughout this period, he remained clinically stable and asymptomatic, with no episodes of transient ischemic attack or stroke. His medical history included renal dysfunction and cardiac pacemaker implantation for arrhythmia, but there was no known history of conditions typically associated with intracranial calcifications.

**Figure 2:**
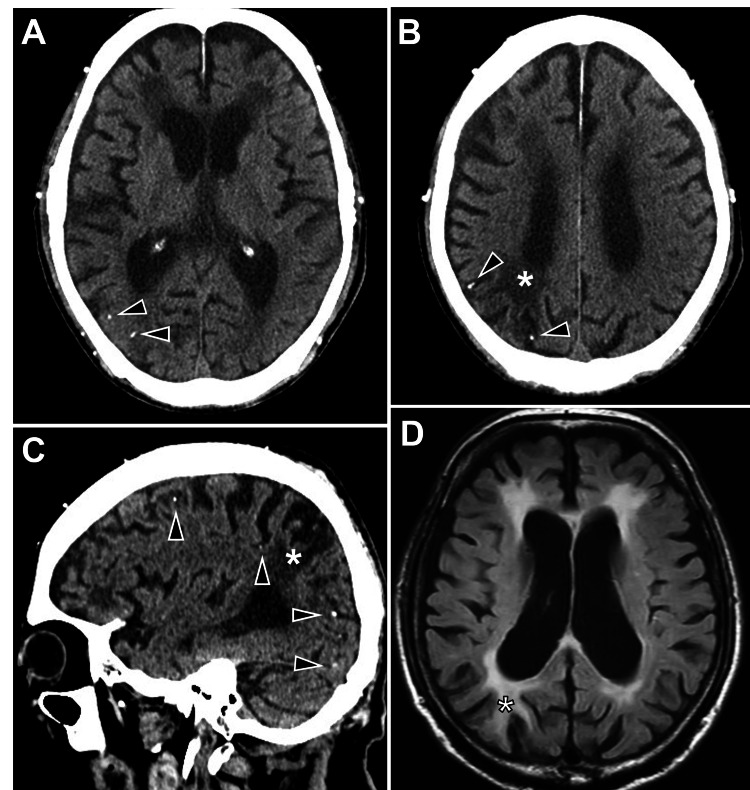
Non-contrast brain CT scans and MRI at the representation Non-contrast brain CT scans demonstrate a low-attenuation region (asterisk) indicative of a chronic infarction in the parieto-posterior watershed area and multiple punctate calcific spots (arrowheads) in the right hemisphere located on the pial surface, exhibiting the characteristic "salted pretzel sign." (A-C) Axial slices. (C) Sagittal slice. (D) Brain MRI shows a chronic infarction in the parieto-posterior watershed area (asterisk). CT: computed tomography: MRI: magnetic resonance imaging

**Figure 3:**
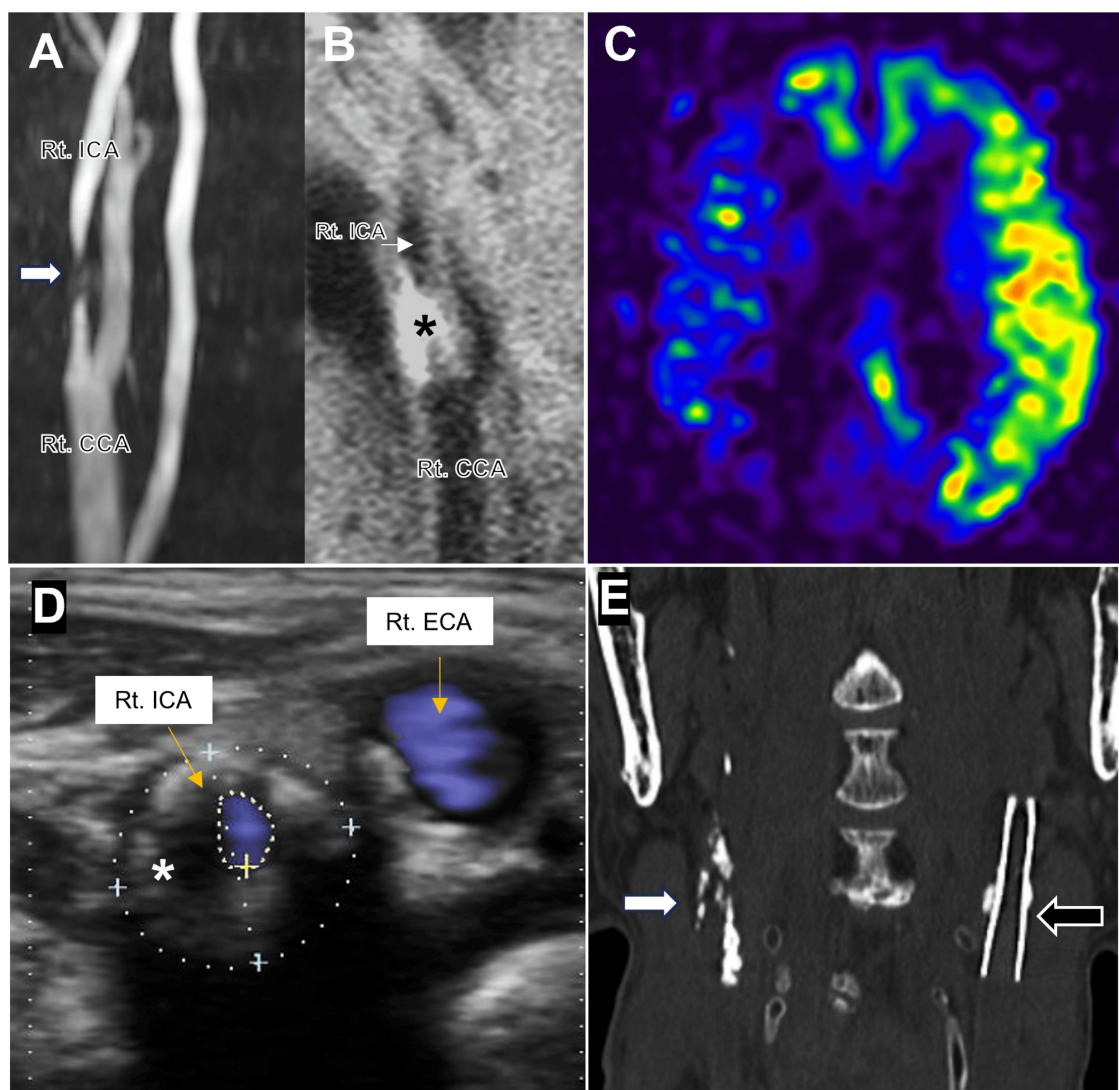
Radiological images at the second intervention (A) Cervical MRA shows severe stenosis with wall irregularity in the right ICA (white arrow). (B) Black-blood T1-weighted image reveals a heterogeneous signal pattern consistent with characteristics of an unstable vulnerable plaque (asterisk). (C) Arterial spin labeling MRI reveals reduced cerebral blood flow in the right hemisphere. (D) Carotid duplex ultrasonography depicts severe luminal stenosis in the right ICA (92% area reduction, peak systolic velocity of 330 cm/second), with a mixed echogenic plaque (asterisk). The blue dotted line outlines the vascular wall, while the yellow dotted line delineates the luminal contour. (E) Coronal slice of the non-contrast cervical CT scan discloses a heavily calcified plaque (white arrow) with scattered small calcifications at the right carotid bifurcation. Stent placement is identified in the left ICA (black arrow). CCA: common carotid artery, ICA: internal carotid artery, ECA: external carotid artery; MRA: magnetic resonance angiography; CT: computed tomography; MRI: magnetic resonance imaging

Five years later, a non-contrast CT scan incidentally showed a low-attenuation region indicative of a chronic infarction in the parieto-posterior watershed area, accompanied by multiple punctate calcifications within the right cerebral hemisphere. These calcifications exhibited the characteristic appearance of the "salted pretzel sign" [5,6] (Figure 2). Notably, these findings were absent on the prior CT scan obtained at the time of the initial intervention (Figure 1B). Subsequent diagnostic evaluation with cervical magnetic resonance angiography (MRA) revealed newly developed significant stenosis with wall irregularity in the right ICA that was absent at the time of the initial intervention (Figure 3A). Black-blood T1-weighted image depicted a mixed-intensity signal, indicating an unstable vulnerable plaque (Figure 3B). Arterial spin labeling magnetic resonance imaging (MRI) revealed reduced cerebral blood flow in the right hemisphere (Figure 3C). Carotid duplex ultrasonography identified severe stenosis with a mixed echogenic plaque at the right carotid bifurcation (92% area reduction, peak systolic velocity of 330 cm/second) (Figure 3D). Additionally, cervical CT imaging confirmed an irregularly and heavily calcified lesion, suggestive of a calcified carotid plaque (Figure 3E). CT angiography and conventional angiography were deemed contraindicated due to underlying renal dysfunction. Comprehensive cardiovascular evaluations revealed no evidence of cardiac pathologies contributing to CCE. Consequently, the multiple punctate calcific lesions were most likely attributed to embolic sequelae originating from the calcified carotid plaque in the right ICA.

**Figure 4:**
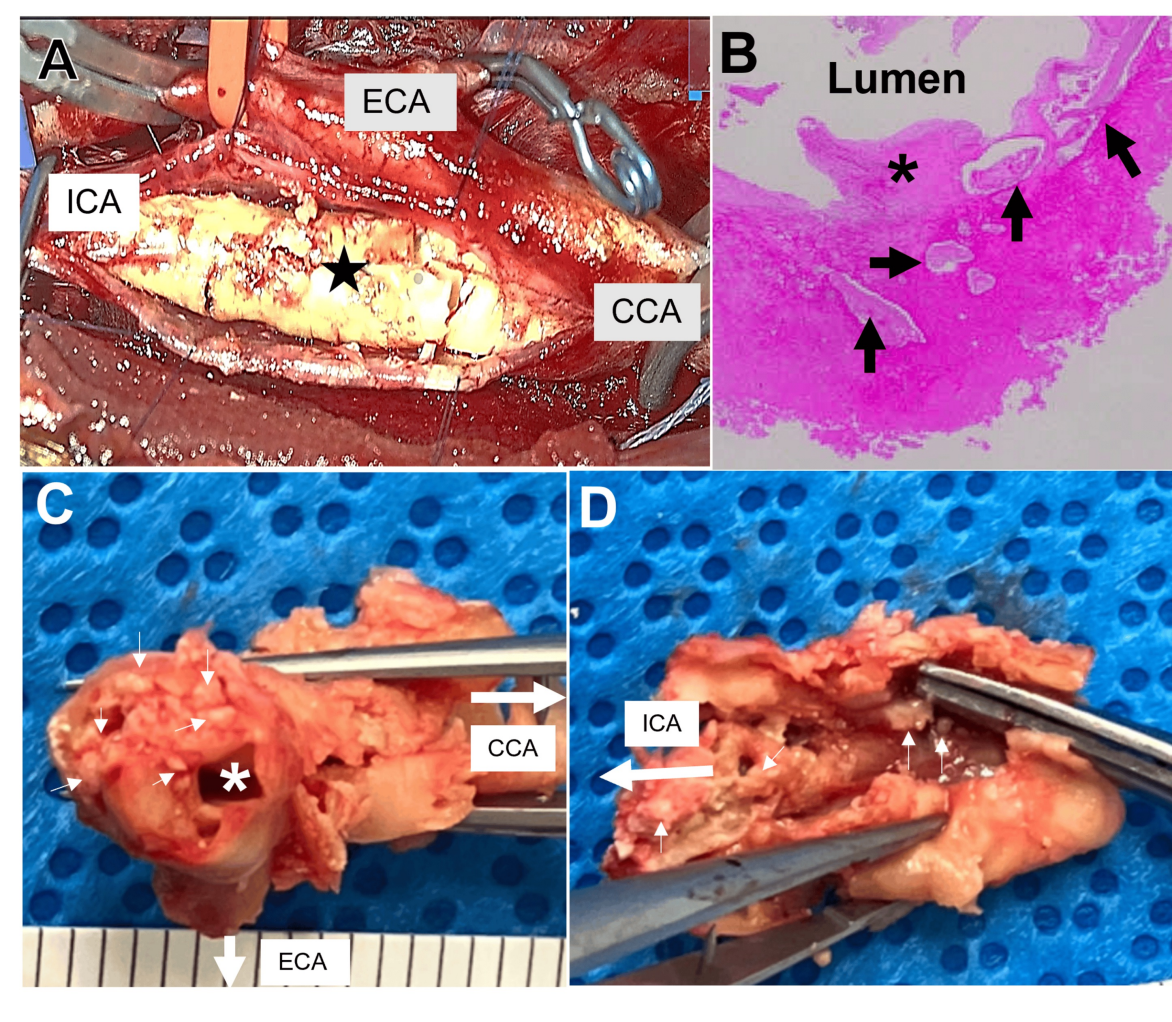
Intraoperative, macroscopic, and histopathological findings of the carotid plaque specimen (A) An intraoperative photograph taken during carotid endarterectomy illustrates the presence of a highly calcified plaque (star). (B) Histopathological image demonstrates a thick intimal layer containing multiple calcific foci (large arrows) and nodular protrusion into the vascular lumen (asterisk). (C) Macroscopic observation of the excised specimen reveals a narrowed vascular lumen (asterisk), with a thick intimal layer containing numerous calcified granules (small arrows). (D) Scattered small calcifications and calcified nodules (small arrows) are observed over the inner surface of vascular lumen. CCA: common carotid artery, ECA: external carotid artery, ICA: internal carotid artery

**Figure 5:**
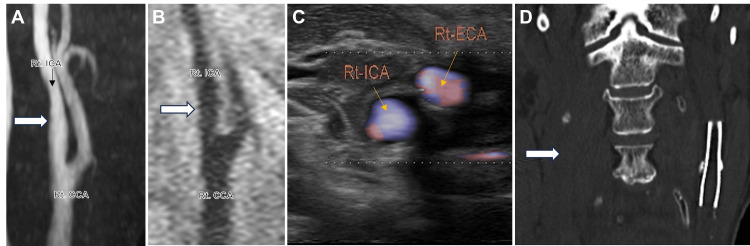
Radiological images after the second intervention (A) Cervical MRA reveals the resolution of the previously noted stenosis in the right ICA (arrow). (B) Black-blood T1-weighted image confirms the disappearance of the previously noted mixed-intensity signal (arrow). (C) Carotid duplex ultrasonography shows the resolution of luminal stenosis of the right ICA with a normalized peak systolic velocity of 60 cm/second. (D) Coronal non-contrast cervical CT reveals the absence of the previously visualized calcified plaque at the right carotid bifurcation (arrow). CCA: common carotid artery, ECA: external carotid artery, ICA: internal carotid artery; MRA: magnetic resonance angiography; CT: computed tomography

Given the characteristic morphology of the unstable carotid plaque, optimal intervention was deemed necessary to mitigate the risk of further embolism. Despite the patient's asymptomatic status, carotid endarterectomy (CEA) of the right ICA was performed successfully. The patient was maintained on oral aspirin (100 mg/day) both before and after surgery. Intraoperatively, a thick intimal layer containing numerous calcified granules causing significant luminal stenosis was identified and excised. Histological analysis of the excised specimen confirmed a heavily calcified plaque with scattered calcium granules and calcific protrusions into the narrowed vascular lumen (Figure 4). In the acute postoperative period, the patient received intensive care, including strict blood pressure control to reduce the risk of cerebral hyperperfusion syndrome and intravenous edaravone (60 mg/day) for neuroprotection. The postoperative course was uneventful, and the patient was discharged with a modified Rankin Scale (mRS) score of 1. Postoperative MRI/MRA and cervical CT confirmed the complete resolution of stenosis with plaque removal, and duplex ultrasound indicated a significant improvement in peak systolic velocity reduced to 60 cm/second (Figure 5). Since the second intervention, the patient has been monitored regularly with MRI and duplex ultrasound. He remains asymptomatic with no evidence of ICA restenosis or recurrence of new cerebral calcified foci, at over one year of follow-up.

## Discussion

Clinical implications of CCE

This case illustrates the clinical importance of identifying CCE, particularly when incidental punctate intracranial calcifications are observed on non-contrast CT scans in asymptomatic patients. In a comprehensive review of 70 cases of CCE, Walker et al. found that only 3% of the patients were asymptomatic with no acute neurological symptoms and that 43% of those with initial CT evidence of CCE later developed recurrent strokes [3]. These findings highlight the rarity of asymptomatic CCE and the clinical importance of accurate recognition and timely therapeutic intervention to reduce the risk of stroke [3,10-12]. Certain individuals, like the present patient, may harbor subtle calcified lesions for years without manifesting overt neurological symptoms, even in the presence of a high-risk carotid plaque burden [3,10-12]. Despite prolonged asymptomatic periods, these lesions indicate an increased risk of future stroke, emphasizing the need for considering preventive treatment strategies [10-12]. 

Diagnostic challenges of CCE

Diagnosing CCE poses a significant challenge due to its subtle radiological manifestations and potential overlap with other intracranial pathologies such as old hemorrhage, vessel wall calcification, or remnants of previous infections [1-4]. In this case, a non-contrast CT scan demonstrated punctate calcific foci along with a low-density area in the watershed zone, consistent with chronic infarction. These findings are of clinical significance, as they not only suggest a previous embolic event but also indicate persistent cerebral ischemia, potentially originating from the stenotic and calcified carotid artery. The "salted pretzel sign" is an important radiological clue, and its accurate recognition can facilitate further diagnostic evaluations to investigate potential embolic sources [5,6]. Cerebrovascular evaluations demonstrated the progression of right ICA stenosis with calcified plaque, implicating a potential source of the embolic foci and cerebral hypoperfusion. Subsequent cardiovascular evaluations revealed no evidence of cardiac pathologies contributing to CCE. This highlights the essential role of comprehensive imaging modalities in accurately diagnosing CCE, thereby delineating the full extent of cerebrovascular and cardiovascular pathologies and guiding optimal therapeutic strategies [3,10-12].

Therapeutic interventions for future stroke prevention

This case emphasizes the importance of considering preventive interventions for patients at risk of recurrent ischemic events, even in the absence of overt neurological symptoms. Carotid artery stenosis, particularly when complicated by calcified plaques, poses an increased risk of ischemic stroke [3,9,13]. Risk factor modification and medical therapy such as antiplatelets, statins, and antihypertensive medications may be effective. Nevertheless, in cases like this, where progressive carotid stenosis and heavily calcified plaque are present, surgical interventions targeting the embolic source can significantly lower the risk [9,13]. This case also highlights the importance of selecting an optimal intervention, particularly when carotid plaques exhibit irregular calcifications. CEA remains a cornerstone of treatment for patients with symptomatic or high-risk carotid artery disease, and its role in preventing embolic events is well established [9,13]. Plaque calcification is reported to be associated with a higher risk of perioperative morbidity and mortality in CAS and an independent risk factor of restenosis following CAS [9,13-15]. Given the extensive calcification in this case, CEA was considered a safer and more effective treatment modality than CAS.

## Conclusions

This case illustrates the clinical significance of recognizing and managing asymptomatic CCE associated with an advanced carotid artery disease. It highlights the rarity of such a presentation and the implications for diagnostic accuracy and stroke prevention. To our knowledge, no prior reports have specifically addressed the diagnosis and management of asymptomatic CCE as described here. A considerable proportion of patients with initial imaging evidence of CCE may be at risk of developing recurrent strokes. A better understanding of the pathophysiology and clinical implications of CCE may contribute to improved outcomes in patients with this uncommon condition.
